# Spatiotemporal analysis of the structure and genetic diversity of *Stemphylium vesicarium* populations in New York onion fields

**DOI:** 10.3389/fmicb.2025.1666712

**Published:** 2025-09-16

**Authors:** Aastha Subedi, Daniel W. Heck, Frank Hay, Natalia Piñeros-Guerrero, Sarah J. Pethybridge

**Affiliations:** ^1^Plant Pathology and Plant-Microbe Biology Section, School of Integrative Plant Science, Cornell AgriTech, Cornell University, Geneva, NY, United States; ^2^Long Island Horticultural Research Laboratory, Plant Pathology and Plant-Microbe Biology Section, School of Integrative Plant Science, Riverhead, NY, United States

**Keywords:** *Allium*, genetic diversity, microsatellites, onion, population biology, population structure, *Stemphylium vesicarium*

## Abstract

Stemphylium leaf blight (SLB), caused by the hemibiotrophic fungus, *Stemphylium vesicarium,* is the dominant foliar disease affecting onions in New York (NY), USA. The development of integrated disease management strategies for SLB is challenged from the lack of information surrounding phylodynamics and evolutionary patterns of the causal organism. This study evaluated the spatiotemporal patterns of genetic diversity, differentiation and population structure of 210 *S. vesicarium* isolates from populations collected over 6 years (2016 to 2022) across five onion production regions in NY using nine microsatellite loci. A total of 158 multilocus genotypes (MLGs) were identified among the 210 isolates, indicating high genetic diversity and genotypic variation. Low genetic differentiation was observed across spatial and temporal populations, with greater genetic variation within populations than between them. Population structure analyses using multiple approaches revealed no clear temporal or spatial genetic patterns, although isolates from 2016 and the Orange County region showed some genetic divergence. Additionally, significant linkage disequilibrium among loci suggested a predominantly clonal population structure in all regions, except the Finger Lakes, which displayed non-significant linkage disequilibrium. These findings highlight the complex population biology and dynamics of *S. vesicarium*, characterized by high genetic diversity, admixture, and mixed reproduction modes and emphasize the challenges in managing SLB, as these characteristics can enable rapid population adaptation to diverse environmental conditions and management practices.

## 1 Introduction

*Stemphylium vesicarium* (Wallr.) E.G. Simmons (Raghavendra Rao and Pavgi, 1975; Woudenberg et al., 2017) is a hemibiotrophic fungal pathogen and causal agent of Stemphylium leaf blight of onion (SLB) (Hay et al., 2021; Sharma and Sharma, 1999). SLB affects onions and other cultivated *Allium* spp. worldwide (Hay et al., 2021; Suheri and Price, 2000, 2001), including more recent reports from Brazil (de Souza Feitosa et al., 2023), Italy (Cortiello et al., 2023), Mauritius (Vally et al., 2024), Mexico (Reyes-Tena et al., 2024), New Zealand (Wright et al., 2019), Slovakia (Horáková et al., 2024), South Korea (Back et al., 2022), Taiwan (Wang et al., 2021) and the Ukraine (Klechkovskyi et al., 2023). *S. vesicarium* also affects other crops such as garlic (Suheri and Price, 2000), leek (Suheri and Price, 2001), asparagus (Graf et al., 2016), and pear (Köhl et al., 2009).

Onion production represents an important component of primary production for New York (NY), comprising approximately 2,400 ha with an annual value of US$63M (USDA NASS, 2024). Most of the intensive onion production in NY occurs on high organic content histosol (muck soils) in Genesee/Orleans Counties (Elba region), and Wayne, Oswego and Orange Counties. The impact of SLB on onion production in northern parts of the U.S.A., and in eastern Canada has escalated in recent years, transforming it from a sporadic threat into a persistent challenge (Hay et al., 2021). Today, SLB is now the most important foliar disease of onion in NY, with epidemics leading to premature plant death and reductions in bulb weight (Hay et al., 2021; Hoepting, 2018; Paibomesai et al., 2012). The emergence of SLB in NY has been associated with the development of resistance to several single site-specific modes of action fungicides which makes disease management increasingly challenging (Hay et al., 2019, 2022a; Pethybridge et al., 2016). Symptoms of SLB begin as water-soaked and pale brown to tan, mottled, oval to spindle-shaped, small lesions (Hay et al., 2021). Older lesions are darker gray or olive-brown in color due to profuse conidial production. As the disease develops, SLB lesions rapidly coalesce and develop into dieback of leaves leading to defoliation (Basallote-Ureba et al., 1999; Hay et al., 2021).

SLB epidemics may be initiated by multiple primary inoculum sources including overwintering inoculum as mycelia, conidia, or pseudothecia and ascospores on infested onion tissue from the previous season, alternative hosts such as weeds (Hay et al., 2021, 2022a, 2022b; McDonald et al., 2023; Stricker, 2021), infected seed (Aveling et al., 1993; Stricker, 2021), volunteer onions surviving from the previous season, or bare-root transplants (Hay et al., 2022b). Secondary spread of SLB within the cropping season results from multiple infection cycles typical of polycyclic disease epidemics (Hay et al., 2022b) resulting from rain and wind-blown dispersal of conidia (Gossen et al., 2021) or onion thrips mediated dispersal (Leach et al., 2020).

Population biology tools offer the opportunity to gather valuable insights into critical aspects of plant disease dynamics, including inoculum sources (Prussin et al., 2013; Zwankhuizen et al., 2000), migration trends (Goss, 2015), phylogeography of the pathogen (Linde et al., 2010), genetic diversity (Rieux et al., 2011), and modes of reproduction (Maciel et al., 2014; Rieux et al., 2011). Understanding the population genetic structure of a pathogen is essential for identifying factors that drive variability including mutations, mating systems, migration, population size, and natural selection (McDonald and Linde, 2002; Milgroom, 2015). Temporal analysis of populations provides insights into evolution and the presence of any genetic bottlenecks, expansions, or shifts in allele frequencies that may occur between growing seasons (Stauber et al., 2022). Spatial analysis can facilitate the identification of geographic structuring and potential barriers to gene flow among regions (Matsuda et al., 2015; Rampersad, 2021). By assessing the genetic diversity and population structure of plant pathogens, effective disease management strategies can be developed, as genetic composition can influence pathogenicity, virulence, and adaptation to environmental conditions (Çelik Oğuz and Karakaya, 2021; McDonald, 2015). Information surrounding the structure of plant pathogen populations is therefore essential to underpin the development of durable integrated disease management strategies to minimize crop loss.

Simple Sequence Repeats (SSRs) or microsatellite markers are a well-established tool to examine the population structure of plant pathogens due to their high polymorphism, co-dominant inheritance, and reproducibility (Jarne and Lagoda, 1996). Their ease of use, low DNA requirements, and affordability make them ideal for studying fine-scale population genetics (Selkoe and Toonen, 2006). Thus, SSR markers have been broadly used to assess genetic diversity, understand population structure, and monitor changes over time and space in pathogen populations (Moges et al., 2016; Rouxel et al., 2012; Widmark et al., 2011). A recent study developed nine SSR markers for *S. vesicarium*, providing essential molecular tools for population genetic studies (Heck et al., 2023). This study utilizes these SSR markers to investigate the spatiotemporal genetic diversity and population structure of *S. vesicarium* across geographically distinct onion production regions in NY over multiple years. By analyzing a larger and more geographically diverse set of isolates, this study aims to uncover patterns of genetic differentiation, admixture, and contribute to a deeper understanding of pathogen persistence and dispersal in agroecosystems, offering valuable information for the development of long-term, sustainable disease management strategies.

## 2 Materials and methods

### 2.1 Sampling and fungal isolations

**Figure 1:**
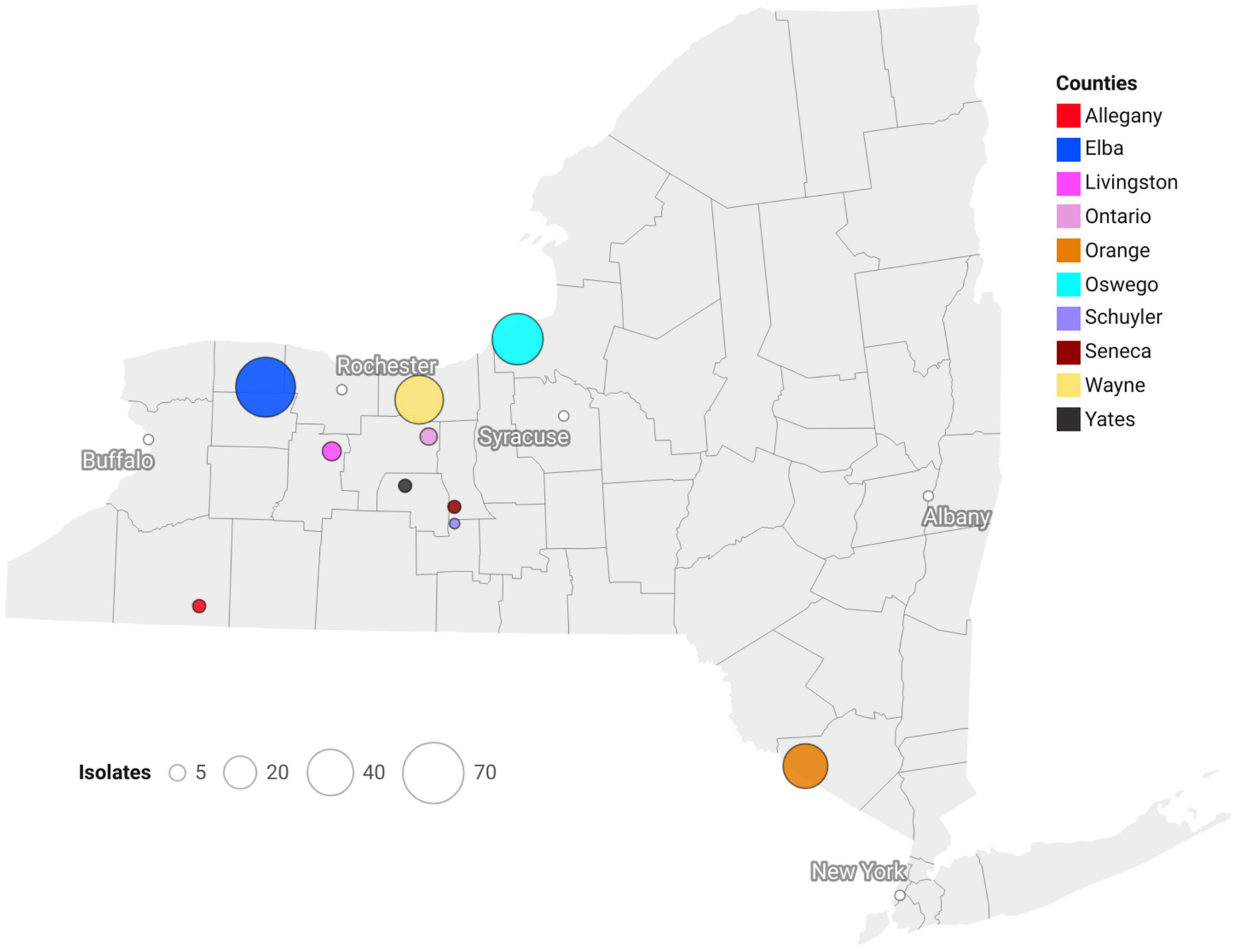
Geographical positions of counties within New York, U.S.A., from where *Stemphylium vesicarium* isolates were collected from onion plants. Population sizes designated by circle diameters.

Isolates of *S. vesicarium* (*N* = 210) were obtained from SLB-affected onion leaves collected in 2016 (*n* = 27), 2018 (*n* = 51), 2020 (*n* = 58) and 2022 (*n* = 74) from production regions across NY (Elba (= Genesse and Orleans Counties): *n* = 67; Orange Co.: *n* = 36; Oswego Co.: *n* = 48; Wayne Co.: *n* = 43; and Finger Lakes (= Allegany, Livingston, Ontario, Schuyler, Seneca, and Yates Cos.): *n* = 16) (Figure 1). One to six isolates were obtained from each field sampled. Due to the similarities and geographical proximity of onion fields from Genesee and Orleans Counties, isolates were grouped and considered as one region (Elba). Elba, Orange, Oswego and Wayne represent the major intensive onion growing regions within NY with production on histosols. Isolates sampled from the Finger Lakes were from fields that consisted of small scale, organic-, and low-input onion production on silt loam soils. The number of isolates was broadly representative of the amount of onion production in each NY region (Supplementary Figure S1 and Supplementary Table S1).

A modified protocol described by Hay et al. (2019) was used to isolate *S. vesicarium* from symptomatic onion leaves. Briefly, samples were examined to confirm the presence of *S. vesicarium* conidia at ×40 magnification. If conidia were not observed, samples were placed in moist chambers (a plastic bag with wet paper towels) for at least 48 h to induce sporulation. Subsequently, 20 μL of sterile distilled water containing 0.01% (v/v) polysorbate 20 (Sigma Aldrich) was placed onto a sporulating lesion and used to dislodge and collect the conidia. The conidial suspension was then spread onto 2% water agar (Hardy Diagnostics) amended with ampicillin (25 mg/L) (Fisher Scientific). Plates were incubated at 25 ± 2 °C for at least 5 h to allow conidia to germinate. A single conidium from each sampling unit (operationally defined as an individual leaf) was then located under the microscope (×40) and removed with a scalpel, transferred onto V8 juice media amended with streptomycin (200 mg/L; Sigma) and incubated at room temperature (25 ± 2 °C) for 6 days. For long-term storage, isolates were grown on synthetic low-nutrient agar (SNA) (Gerlach and Nirenberg, 1982) for 10 days and colonized plugs were preserved in sterile distilled water and kept at 25 ± 2 °C (Castellani, 1963). Isolates were also preserved in 30% glycerol and stored at −80 °C (Heckly, 1978) and included in the fungal collection of the Epidemiology of Vegetable Diseases Laboratory of Cornell AgriTech, Geneva, NY, U.S.A.

### 2.2 DNA extraction

Isolates were retrieved from long-term storage and grown on V8 agar media at 25 ± 2 °C for 10 days. Fungal mycelia were harvested and dried overnight in a laminar flow cabinet. Genomic DNA was extracted from all 210 isolates using the Wizard Genomic DNA Purification Kit (Promega) following the manufacturer’s recommended protocol. DNA concentration was quantified using Nanodrop Spectrophotometer ND-1000 (NanoDrop Technologies). The final concentration of DNA was adjusted to 25 ng/μL for all multiplex PCR assays.

To confirm species identity, a subsample of 74 isolates were amplified with the oligonucleotides set KES1999 and KES2000 (Graf et al., 2016) developed to qualitatively differentiate *S. vesicarium* and *S. botryosum* based on amplicon size. All 74 isolates were identified as *S. vesicarium*, with an amplicon of ~420 bp in size (*data not shown*). Additionally, the isolates On16-63 and On16-381 used in this present study had previously undergone whole genome sequencing (Sharma et al., 2020).

### 2.3 Genotyping of *S. vesicarium* populations

Nine SSR markers, previously developed for *S. vesicarium* were used to genotype all 210 *S. vesicarium* isolates. These markers were organized into two multiplex PCR assays (Heck et al., 2023). Each multiplex PCR was performed in a 12.5 μL reaction volume containing 2.5 μL of Multiplex 5 × Master Mix (New England Biolabs), variable volumes of each labeled primer pair, 1 μL of template DNA (25 ng), and ultrapure autoclaved water to reach the final volume. PCR assays were conducted in a T100™ thermal cycler (Bio-Rad) following an initial denaturation at 95 °C for 5 min; 35 cycles of 30 s at 95 °C, 30 s at 57 °C, and 30 s at 68 °C; followed by a final extension at 68 °C for 5 min. The quality and expected size of PCR products were verified by electrophoresis on a 3% (w/v) agarose gel.

Amplified fragments were analyzed at the Cornell Institute of Biotechnology Facility (Cornell University) using an ABI 3730xl sequencer with a GS-500LIZ size standard dye (Applied Biosystems). Chromatograms were processed in Geneious software (ver. 7.1.7; Biomatters Ltd.) using the Microsatellite 1.4.7 plugin (Kearse et al., 2012). A minimum call rate of 0.1 was set per locus, allowing up to 10% missing data per locus per isolate. Poor quality sequences were reanalyzed as needed. Population genetic analyses were structured into two strata: (1) four temporal populations based on sampled years; and (2) five spatial populations based on geographic location. A subset of 32 isolates spanning three of the four temporal populations and all five geographical locations of all nine SSR’s were genotyped up to three times for reproducibility evaluation. In total, 864 data points were obtained, with a reproducibility rate of 0.9826, where 15 data points failed to produce signaling peaks (10) or had a different size than expected (5).

### 2.4 Data analyses

#### 2.4.1 Locus summary statistics

The *locus_table* function within poppr package version 2.9.3 (Kamvar et al., 2014) was used to calculate the number of alleles per locus (*N_a_*), Simpson’s index (1-D) (Simpson, 1949), Nei’s allelic diversity (*H_e_*; Nei, 1978), and evenness (*E_5_*; Ludwig and Reynolds, 1988). Noninformative loci were identified using the *informloci* function with the default cutoff value in the *poppr* package version 2.9.3 in the R statistical software (Kamvar et al., 2014). To evaluate whether the SSR loci provided sufficient variability to distinguish unique *S. vesicarium* isolates, a genotype accumulation curve was constructed with 1,000 resamples without replacement within the *genotype_curve* function in *poppr*.

#### 2.4.2 Genotypic diversity indices

Genotypic diversity, multilocus genotypes (MLGs) counts and Nei’s expected heterozygosity (*H_exp_*) were computed for each population using *poppr* (Kamvar et al., 2014). To account for potential biases due to unequal sample sizes across years and geographic locations, diversity indices, including Shannon–Wiener’s index (*H′*; Shannon, 1948), Simpson’s genotypic diversity index (*λ*; Simpson, 1949), Stoddart and Taylor’s genotypic diversity index ( $\hat{G})$ (Stoddart and Taylor, 1988), and evenness (*E₅*) were estimated using rarefaction. This rarefaction was applied to the smallest population sample sizes (26 for temporal populations and 16 for spatial populations) and conducted with 1,000 jackknife replicates in *poppr* (Kamvar et al., 2014).

#### 2.4.3 Linkage disequilibrium

Linkage disequilibrium (LD) for each population was assessed using the standardized index of association ( $\bar{r}$ _D_) under clone correction in *poppr* (Agapow and Burt, 2001). Statistical significance of LD was tested with 999 permutations, where the null hypothesis assumed no LD (i.e., alleles at different loci are unlinked and randomly associated within populations).

#### 2.4.4 Population differentiation

Analysis of molecular variance (AMOVA) was conducted using clone corrected data implemented in *poppr* (Kamvar et al., 2014) to estimate the proportion of genetic variation within and among populations. The significance of the AMOVA was tested with 9,999 permutations. Genetic differentiation between population pairs was evaluated by calculating the pairwise Jost’s *D* differentiation index (Jost, 2008) using the *mmod* ver. 1.3.3 package (Winter, 2012) in R studio version 4.4.1 (R Core Team, 2024). Statistical significance of Jost’s *D* was determined through a randomization test, where all isolates within the populations were randomly reassigned, and Jost’s *D* was recalculated from the randomized dataset. This test was performed under the null hypothesis of no genetic differentiation between populations.

#### 2.4.5 Population structure

The genetic structure in temporal and spatial populations was analyzed using multiple approaches. First, discriminant analysis of principal components (DAPC), a multivariate clustering method, was applied to display genetic clustering while minimizing within-cluster variation. This analysis was conducted using the *adegenet* package v. 2.1.2 (Jombart, 2008) in R studio version 4.4.1 (R Core Team, 2024). Non-clone corrected data from temporal and spatial populations were separately transformed into principal components (PCs), with the optimal number of PCs (temporal = 19 PCs; spatial = 40 PCs) determined by cross-validation. The proportion of conserved variance for temporal and spatial populations were 93 and 78%, respectively. Next, a Bayesian model-based clustering analysis was performed using STRUCTURE ver. 2.3.4 (Pritchard et al., 2000), which assigns individuals to genetic clusters based on allele frequencies at each locus. Sampling years and geographic locations were incorporated as prior information (LOCPRIOR), along with the admixture model and correlated allele frequencies were selected for the analysis. Each analysis included a burn-in of 100,000 iterations followed by an additional 100,000 Markov Chain Monte Carlo iterations, across 10 independent runs for each value of K (1 to 20 clusters). The optimal K was identified by evaluating the ΔK method of Evanno et al. (2005) using STRUCTURE SELECTOR (Li and Liu, 2018). To further explore genetic relationships among populations, an Unweighted Pair Group Method with Arithmetic Mean (UPGMA) and a neighbor-joining (NJ) tree was constructed using pairwise Nei’s genetic distance matrix (Nei, 1978) in *poppr* (Kamvar et al., 2014). Finally, a minimum spanning network (MSN) was constructed to assess the genetic relatedness among MLGs in populations both at temporal and spatial scales using the *imsn* function in *poppr* based on Bruvo’s genetic distance (Bruvo et al., 2004) in the *adegenet* R package (Jombart, 2008).

## 3 Results

### 3.1 Locus summary statistics

The number of alleles (*N_a_*) per locus ranged from 3 to 41, with an average of 12.56 alleles per locus. Simpson index (1-*D*) ranged from 0.26 to 0.94, with an average of 0.66. Nei’s gene diversity (*H_e_*) ranged from 0.26 to 0.95, with an average of 0.66, and evenness (*E_5_*) ranged from 0.48 to 0.83, with an average of 0.68. The locus SvSSR09 had the highest number of alleles (*N_a_* = 41), and allelic diversity based on *H_e_* (0.95), while SvSSR15 was the least diverse, with *N_a_* = 3 and *H_e_* = 0.52 (Supplementary Table S2). None of the loci were found to be uninformative. The genotypic accumulation curve reached a plateau at 158 MLGs from a total of 210 isolates (Supplementary Figure S2).

### 3.2 *Stemphylium vesicarium* population genotyping

Of the 210 isolates included in the study, 158 unique MLGs (75%) were identified within the entire *S. vesicarium* population. With the varying number of samples, the number of MLGs also varied temporally and spatially across populations. Across years, the populations sampled in 2016, 2018, 2020, and 2022 contained 26, 46, 41, and 60 MLGs, respectively (Table 1). Across the locations, 30, 40, 15, 46, and 43 MLGs were observed in the Orange, Wayne, Finger Lakes, Elba, and Oswego populations, respectively (Table 1). Few MLGs (8%) were shared between spatially distinct populations, suggesting local adaptation. The expected multilocus genotype (*eMLG*) values further supported variability, with more *eMLGs* observed across years (mean = 25) than location (mean = 15). For temporal populations, *eMLGs* ranged from 21.47 in the 2020 population to 26 in the 2016 population. Across locations, *eMLGs* ranged from 13.7 in Elba to 15.51 in Wayne (Table 1).

**Table 1:** Genetic diversity indices for *Stemphylium vesicarium* populations collected from onion fields in multiple production regions within New York, U.S.A., from 2016 to 2022.

Strata	Pop^a^	*N* ^b^	MLGs^c^	*eMLG* ^d^	SE^e^	*H* ^f^	*G* ^g^	λ^h^	*E_5_* ^i^	*H_exp_* ^j^
Temporal	2016	27	26	26.000	0.000	3.244	25.138	0.960	0.979	0.734
	2018	51	46	25.489	0.994	3.215	24.175	0.958	0.967	0.636
	2020	58	41	21.474	1.692	2.965	17.122	0.940	0.864	0.630
	2022	74	60	24.526	1.356	3.157	22.326	0.955	0.942	0.658
	Total	210	158	25.109	1.351	4.858	88.200	0.989	0.682	0.664
Spatial	Orange	36	30	14.633	0.974	2.641	13.509	0.925	0.952	0.467
	Wayne	43	40	15.514	0.648	2.731	15.163	0.934	0.984	0.683
	Finger Lakes	16	15	15.000	0.000	2.686	14.222	0.930	0.967	0.756
	Elba	67	46	13.701	1.329	2.549	12.047	0.914	0.919	0.661
	Oswego	48	43	15.468	0.660	2.727	15.088	0.933	0.984	0.641
	Total	210	158	15.295	0.842	4.858	88.200	0.989	0.682	0.664

a Populations (Pop) of *S. vesicarium* based on temporal and spatial strata.

b Number (N) of isolates.

c Multilocus genotypes (MLGs).

d Number of expected MLGs (*eMLGs*) at the smallest sample size based on rarefaction.

e Standard error (SE) based on rarefaction.

f Shannon–Wiener (H′), genotypic diversity after rarefaction (Shannon, 1948).

g Stoddart and Taylor’s ( $\hat{G}$ ), genotypic diversity after rarefaction (Stoddart and Taylor, 1988).

h Simpson’s (λ) diversity index after rarefaction (Simpson, 1949).

i Evenness (*E*_5_) after rarefaction (Ludwig and Reynolds, 1988).

j Nei’s expected heterozygosity (H_exp_; Nei, 1978).

The entire *S. vesicarium* population exhibited high genetic diversity, with genotypic diversity indices after rarefaction indicating temporal variation. For example, diversity was generally higher in populations sampled in 2016 (Table 1). Among spatially distinct populations, Wayne and Oswego had the highest rarefied genotypic diversity based on indices *H′*, $\hat{G}$ , and *λ*, while the Elba population was the least diverse. Genotypic evenness (*E_5_*) was consistently high (> 0.86) for both temporal and spatial populations, indicating an even distribution of genotypes. The average expected heterozygosity (*H_exp_*) also varied across populations. Across locations, *H*_*ex*p_ ranged from 0.47 to 0.76 in Orange and Finger Lakes populations, respectively. The lowest genetic diversity was found in the population from 2020 (*H_exp_* = 0.63), while the 2016 population had the highest genetic diversity (*H_exp_* = 0.73; Table 1).

### 3.3 Linkage disequilibrium

The overall standardized index of association for the clone corrected population ( $\bar{r}$ _D_ = 0.05) was significantly different from zero (*p* < 0.001; Table 2). This significant departure indicates a non-random association of alleles across loci, suggesting the population is in linkage disequilibrium. When examining linkage disequilibrium between different years and geographic populations individually, significant linkage disequilibrium (*p* > 0.05) was observed in all populations except for the Finger Lakes ( $\bar{r}$ _Dcc_ *=* 0.007, *p* = 0.323; Table 2).

**Table 2:** Indices of association for *Stemphylium vesicarium* populations from onion fields in multiple production regions within New York, U.S.A., from 2016 to 2022.

Strata	Populations	I_A_^a^	$\bar{r}$ _D_^a^	I_ACC_^b^	$\bar{r}$ _D CC_ ^b^
Temporal	2016	0.759^*^	0.100^*^	0.705^*^	0.093^*^
	2018	0.530^*^	0.069^*^	0.519^*^	0.067^*^
	2020	0.942^*^	0.121^*^	0.576^*^	0.075^*^
	2022	0.535^*^	0.069^*^	0.467^*^	0.060^*^
	Total	0.497^*^	0.065^*^	0.419	0.055^*^
	Orange	0.562^*^	0.074^*^	0.534^*^	0.071^*^
	Wayne	0.425^*^	0.055^*^	0.383^*^	0.050^*^
Spatial	Finger Lakes	0.274	0.036	0.054	0.007
	Elba	0.965^*^	0.124^*^	0.648^*^	0.084^*^
	Oswego	0.353^*^	0.046^*^	0.252^*^	0.033^*^
	Total	0.497^*^	0.065^*^	0.401^*^	0.052^*^

a I_A_ and $\bar{r}$ _D_ indices of association without clone correction.

b I_ACC_ and $\bar{r}$ _DCC_ indices of association after clone correction.

* Indicates populations in linkage disequilibrium (*p* < 0.01).

### 3.4 Population differentiation

Most of the genetic variation was found within populations, accounting for 98.5 and 95% of the total variation in temporal and spatial populations, respectively (Table 3). In contrast, only a small proportion of the variation was attributed to differences between populations, with 1.5% observed for temporal populations (ΦST = 0.015, *p* = 0.002) and 5% for spatial populations (ΦST = 0.048, *p* = 0.001).

**Table 3:** Analysis of molecular variance (AMOVA) of the clone-corrected datasets of *Stemphylium vesicarium* populations from onion fields in multiple production regions within New York, U.S.A., from 2016 to 2022.

Strata	Variations	*df*	Sum of squares	Mean square	Variation (%)	*P =*
Temporal	Between populations	3	29.95	9.98	1.53	0.002
	Within populations	169	1020.41	6.04	98.47	
	Total	172	1050.36	6.11		
Spatial	Between populations	4	63.53	15.88	4.82	0.001
	Within populations	169	988.83	5.85	95.18	
	Total	172	1052.36	6.08		

The coefficient of genetic differentiation (Jost’s *D*) revealed a low degree of differentiation between *S. vesicarium* populations. When populations were stratified temporally, Jost’s *D* values ranged from 0.018 to 0.098, with the 2016 population showing a marginally higher level of differentiation (Table 4). All pairwise comparisons between temporal populations were significant except between the 2018 to 2020 population. Similarly, spatial stratification also showed low pairwise genetic differentiation, with Jost’s *D* values between 0.036 and 0.23, with the exception of higher Jost’s *D* values identified in the Orange population (Table 4). The magnitude of these Jost’s *D* values is relatively low, however, most of the significant values suggest that while there was some level of genetic differentiation among the *S. vesicarium* populations the overall differentiation was not strong.

**Table 4:** Pairwise comparison of population differentiation using Jost’s *D* between *Stemphylium vesicarium* populations collected from onion fields in multiple production regions within New York, U.S.A., from 2016 to 2022.

Strata	Populations	2016	2018	2020
Temporal	2016			
	2018	0.098^*^		
	2020	0.097^*^	0.018	
	2022	0.087^*^	0.053^*^	0.053^*^

	Populations	Orange	Wayne	Finger Lakes	Elba
Spatial	Orange				
	Wayne	0.143^*^			
	Finger Lakes	0.225^*^	0.080		
	Elba	0.234^*^	0.073^*^	0.061	
	Oswego	0.103^*^	0.036	0.137^*^	0.127^*^

* Indicates significant differentiation between populations (*p* < 0.01).

### 3.5 Population structure

**Figure 2:**
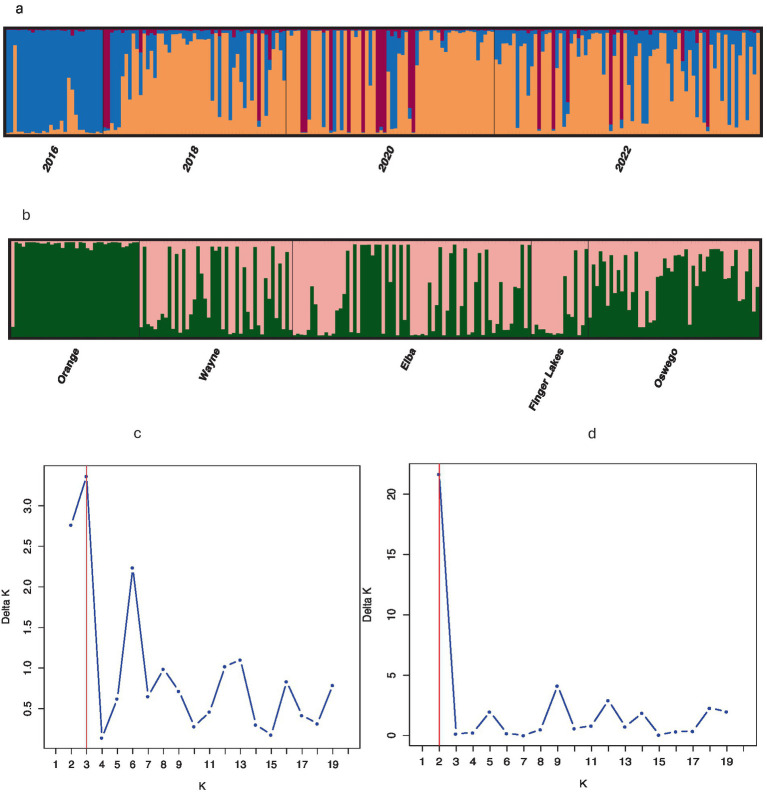
Population structure and ΔK plot estimated by STRUCTURE of 210 *Stemphylium vesicarium* isolates using nine simple sequence repeat loci. Each color denotes a population determined by the analysis, divided into *K* colors, where *K* is the number of clusters assumed. Each isolate is represented by a bar and the height of each column represents the probability of membership to each cluster according to **(a)** year of sampling, *K* = 3 **(b)** geographic region across years, *K* = 2 **(c)** ΔK plot at temporal scale; and **(d)** ΔK plot at spatial scale.

**Figure 3:**
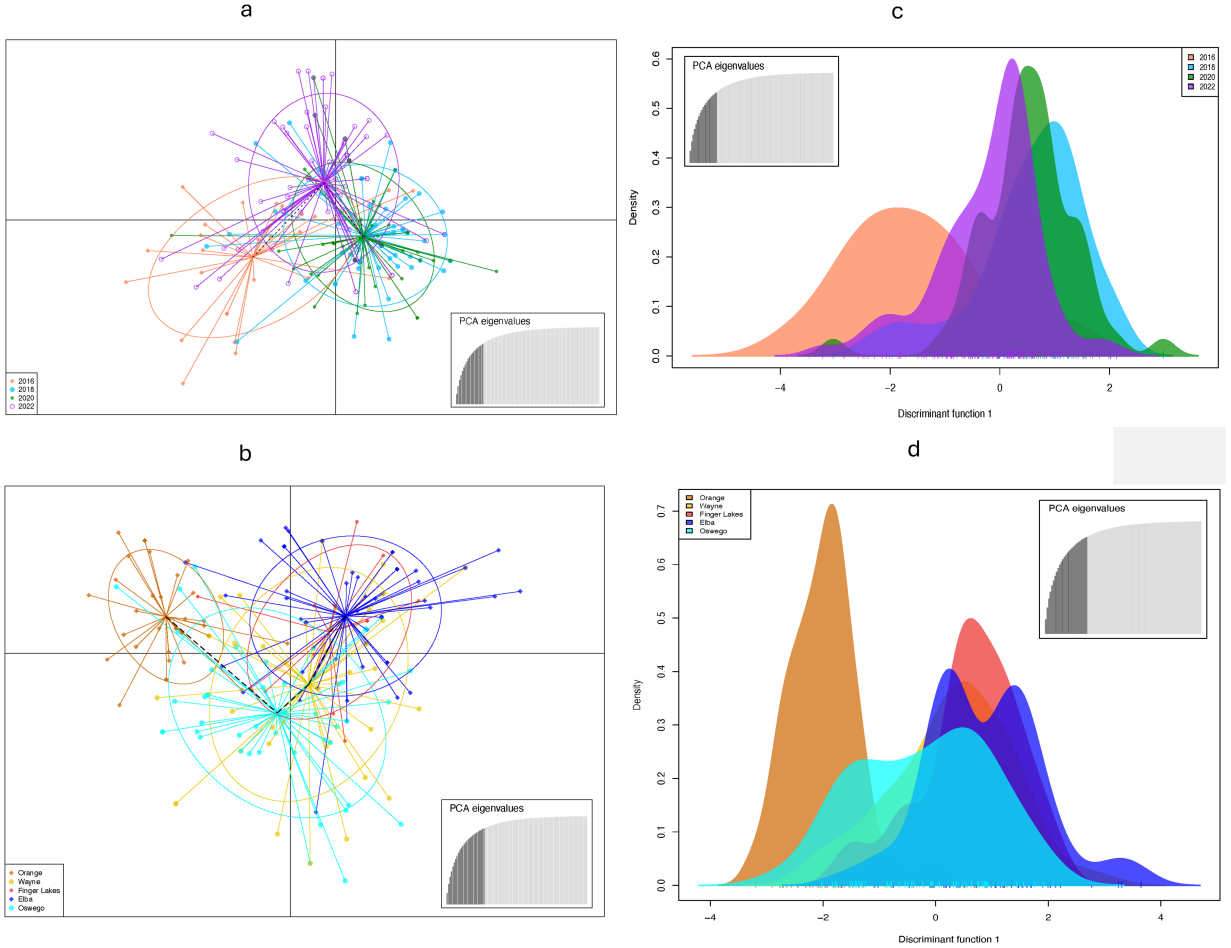
Discriminant analysis of principal components (DAPC) using nine simple sequence repeats to characterize *Stemphylium vesicarium* populations from onion fields across New York, U.S.A. **(a,b)** Genetic differentiation among **(a)** temporal populations **(b)** spatial populations by DAPC. Each population are displayed by different colors. Dots represent individual isolate. **(c,d)** Density plot of individuals along the first discriminant function from the DAPC for **(c)** temporal populations **(d)** spatial populations.

**Figure 4:**
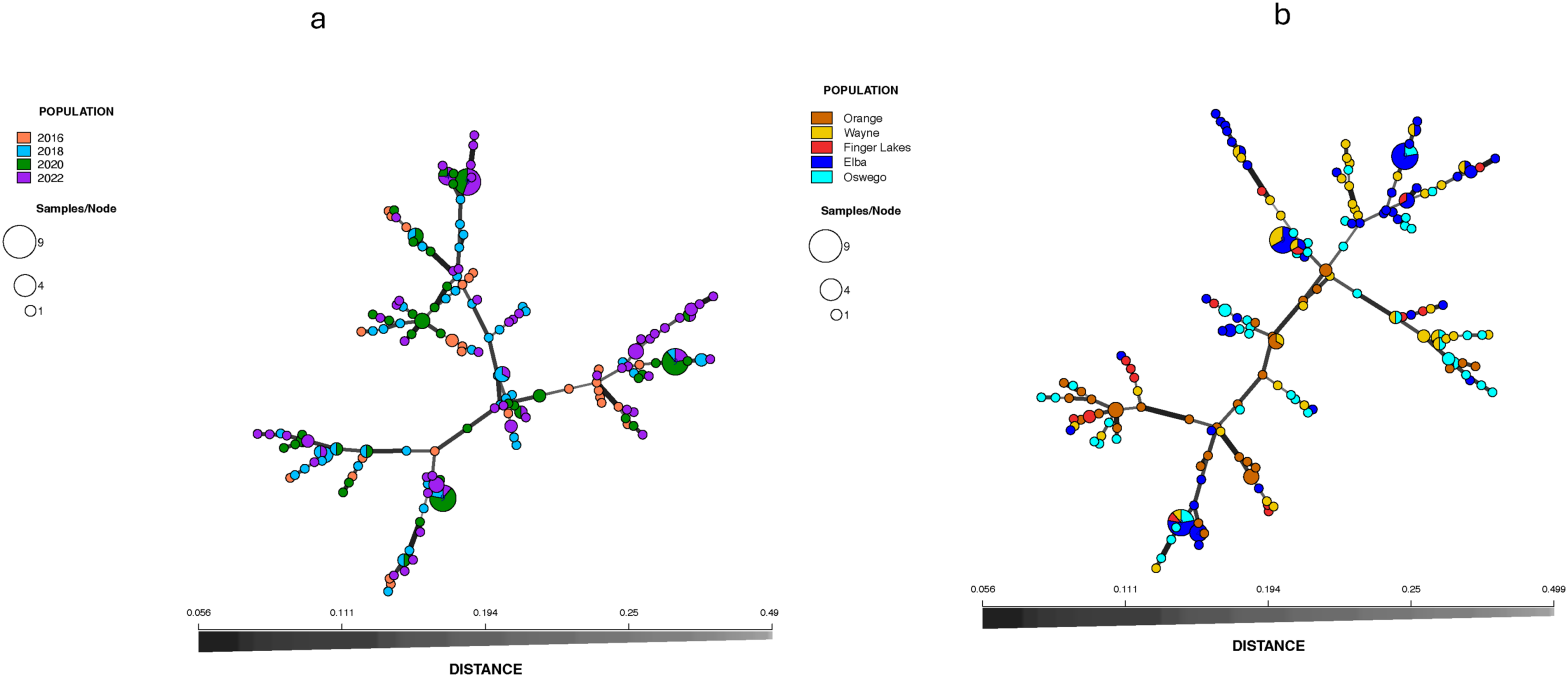
Minimum spanning network (MSN) showing the relationships among individual multilocus genotypes (MLGs) characterized within *Stemphylium vesicarium* populations from onion fields across New York, U.S.A., according to **(a)** year (2016 to 2022), and **(b)** multiple production regions over years. Each node (circle) represents an MLG. Distances between nodes are proportional to Bruvo’s genetic distance. Node colors represent population membership, and node sizes correspond to the number of isolates.

Population structure analysis identified three genetic clusters across 4 years, with the 2016 population primarily associated with one cluster (denoted blue in Figure 2a), as determined by the largest ΔK value of 3.75 found at K = 3 (Figure 2c). The 2018, 2020, and 2022 populations were mostly associated with a separate cluster (denoted orange in Figure 2a) and displaying admixture with two clusters (denoted blue and brown in Figure 2a). When grouped by location, the ΔK value (= 22) reached a sharp peak at K = 2 (Figure 2d) revealing two clusters, with the Orange population showing almost no admixture compared to significant levels of admixture in other locations (Figure 2b). DAPC analysis, retaining optimal PCs, confirmed weak associations between clustering and years or locations, although isolates from Orange County or sampled in 2016 showed slight tendencies to form distinct clusters (Figure 3), consistent with results from the population structure analysis. Furthermore, the MSN displayed no clear clustering of genotypes by year or location. Isolates from all temporal and spatial groups were scattered across various branches of the network, although isolates from the Orange population were somewhat more concentrated towards the center and lower portions of the network (Figure 4). The UPGMA tree defined three temporal clusters (2016, 2018/2020, and 2022; Supplementary Figure S3a) and three spatial clusters (Orange, Elba/Finger Lakes, and Oswego/Wayne; Supplementary Figure S3b). Based on a 75% bootstrap threshold, the temporal clustering (Supplementary Figure S3a) shows relatively strong support for the three temporal groups, while the spatial clustering (Supplementary Figure S3b) exhibits low bootstrap values for most clusters suggesting weak support, with the exception of Oswego + Wayne (82.7). Analyzing all 18 populations together across different years and locations also did not reveal distinct clustering patterns based solely on year or location, although the population from Orange in 2016 and Finger Lakes in 2018 (bootstrap value = 97.4) showed substantial differentiation (Supplementary Figure S3c).

## 4 Discussion

*Stemphylium vesicarium* populations from NY onion fields exhibited high genetic diversity with minimal spatial and temporal genetic structure. The analysis of nine SSR loci identified 158 distinct MLGs (75%) of 210 isolates, indicating high genotypic richness. The temporal fluctuations in *eMLG*s and genetic diversity indices suggested shifts in population structure potentially driven by environmental pressures or management practices. For example, the 2016 isolates tended to form a distinct genetic cluster indicating some divergence. Moreover, the observed admixture in *S. vesicarium* populations from 2018, 2020, and 2022, suggests ongoing gene flow and genetic mixing over time.

Similarly, the spatial distribution of *eMLG*s, along with comparatively high genetic diversity, particularly in Wayne and Oswego, suggest localized adaptation, where populations may be evolving at a different rate and/or in response to distinct agricultural practices in these regions. Although population structure identified fewer clusters (K = 2) than the five spatial populations analyzed, significant structuring was not present. Based on multiple population structure analysis, the Orange population showed some degree of genetic isolation with almost no admixture, while all other populations showed significant admixture, indicative of common source or gene flow among regions. Furthermore, UPGMA clustering grouped Elba and Finger Lake populations, and Oswego and Wayne populations within an additional distinct cluster. The Elba and Finger Lakes regions are geographically closer to each other but have distinctly different management strategies. The Finger Lakes region mostly consists of geographically isolated, small-scale, organic or low-input farms, on mineral soils. Conversely, the Elba region is composed of several large-scale conventional farms located on a pocket of histosol soil using high inputs and with fields located near each other. Wayne and Oswego counties are closer to each other, and differentiate from Elba, by having farms located on several smaller and geographically separate areas of histosoils. Orange County production is akin to Elba with multiple onion fields and farms neighboring each other, in a single, large area of histosols soil, and geographically distant from other regions in the study. *S. vesicarium* populations analyzed using amplified fragment length polymorphism markers also showed no evidence of spatial association between genotypes (Köhl et al., 2009).

The genetic variability in *S. vesicarium* populations from NY onion fields was therefore predominantly attributed to within populations, with minimal differentiation between populations. The 2016 population exhibited slightly significantly higher differentiation (Jost’s *D* = 0.087–0.098), likely to reflect initial genetic variation homogenized through gene flow or the influence of agronomic selection pressures (i.e., fungicide applications) in the subsequent years. The Orange population displayed a marginally higher significant Jost’s *D* value (0.10–0.23), suggesting localized genetic variation potentially driven by unique environmental conditions or specific management practices in that area. Low genetic differentiation implies that there is likely high gene flow among the populations, which can homogenize their genetic composition over time, possibly due to frequent migration events or evolutionary processes such as random genetic drift and mutation rates (Adhikari et al., 2021; Zhan, 2016).

Linkage disequilibrium analysis revealed significant non-random associations of alleles across loci in all populations except the Finger Lakes, which includes more organic fields compared to other regions, suggesting predominantly clonal reproduction. The reproductive biology of *S. vesicarium* is characterized by homothallism allowing self and outcrossing (Inderbitzin et al., 2005). Homothallic species like *S. vesicarium* can undergo syngamy between genetically identical haploid cells, resulting in haploid selfing and producing a population structure akin to clonal reproduction (Billiard et al., 2012). Conversely, outcrossing during sexual reproduction generates a recombining population structure (Milgroom, 1996; Smith et al., 1993; Tibayrenc et al., 1991). However, it remains unclear whether clonal genotypes arise solely from selfing, asexual reproduction, or a combination. While analyzing ascospore populations could help clarify the contribution of sexual reproduction, the homothallic nature of the pathogen makes controlled crosses challenging, leaving the role of outcrossing in natural populations unresolved (Attanayake et al., 2014). The non-significant linkage disequilibrium in the Finger Lakes population may suggest higher recombination, but its smaller sample size warrants caution in interpretation. Outcrossing through sexual reproduction is often linked to high genetic diversity due to recombination and the generation of novel genotypes (Atallah et al., 2004; Bihon et al., 2012; Brewer et al., 2015; McDonald, 1997; Milgroom, 1996). The significant linkage disequilibrium in populations with high genetic diversity in this study may seem counterintuitive. However, studies have shown that both in homothallic (Brewer et al., 2015) and heterothallic (Gañán-Betancur et al., 2021) pathogens, significant linkage disequilibrium can occur alongside high genetic diversity. Moreover, sampling timing could influence linkage disequilibrium interpretation. Samples in this study were collected later in the season when approaching harvest, when polycyclic asexual reproduction dominates and could bias results toward clonal structures of the population. Future studies should consider sampling at different time points in the growing season to better capture the full spectrum of reproductive modes of *S. vesicarium*.

The high genetic diversity of *S. vesicarium* may be attributed to factors such as importation of infected transplants from other states, and long-range dispersal of ascospores from pseudothecia produced in overwintering substrates like infested plant debris, alternative hosts including weeds, or volunteer onion plants (Gossen et al., 2021; Hay et al., 2021; McDonald et al., 2023). The significant role of external inoculum sources in SLB spread, suggested by the lack of significant spatiotemporal association (Hay et al., 2022b), likely drives the emergence of new genotypes, thereby sustaining continuous genetic diversity without clearly defined genetic structure. The high genotypic diversity may result from multiple introductions and subsequent population admixture, as supported by the population structure analyses. A similar pattern was observed in *Alternaria brassicicola* where multiple founder populations, resulting in the admixture, drove high gene and genotypic diversity (Linde et al., 2010). Additionally, high dispersal potential through airborne spores (Basallote-Ureba et al., 1999; Gossen et al., 2021; Katoch and Kumar, 2017; Simmons, 1969), insects (Leach et al., 2020), and the movement of infected planting material may facilitate genetic mixing over time and space. The lack of strong genetic structure implies uniform adaptation of the pathogen across locations and years, complicating management strategies.

Additional population genetic analyses, including *S. vesicarium* populations from other onion-growing regions in the U.S. and worldwide, could shed light on the pathogen’s dispersal patterns and invasion pathways. The coexistence of organic and conventional farms and varying host susceptibility levels, also likely influence the evolutionary dynamics of *S. vesicarium*. Future studies integrating host cultivar information, inoculum sources, and the role of infested onion transplants are crucial for a comprehensive understanding of genetic diversity and patterns. Overall, these findings highlight the need for integrated management strategies that account for regional genetic dynamics and monitoring genetic changes over time to effectively address pathogen adaptation and dispersal. Addressing the unresolved questions about the pathogen’s reproductive strategies and dispersal mechanisms will be key to mitigating the impact of *S. vesicarium* on onion production. This study provides valuable insights into the population biology of *S. vesicarium*, an economically important foliar pathogen affecting onion production in NY. Future studies should integrate advanced genomic tools, epidemiological data, and *S. vesicarium* populations from diverse regions, while considering management factors, reproductive strategies, and dispersal mechanisms, to better understand the pathogen’s evolution and inform comprehensive, adaptive disease management strategies.

## Data Availability

The original contributions presented in the study are included in the article/Supplementary material, further inquiries can be directed to the corresponding author.
